# Tailored strategies for improved control of CAR-T cells in multiple myeloma

**DOI:** 10.3389/fimmu.2026.1740345

**Published:** 2026-01-29

**Authors:** Anna Bielowski, Teresa Kilian, Sarah Vera-Cruz, Quentin Deveuve, Sabrina Kraus, Hermann Einsele, Michael Hudecek, Sophia Danhof

**Affiliations:** 1 Chair for Cellular Immunotherapy, Department of Internal Medicine II, University Hospital Würzburg, Würzburg, Germany; 2 Department of Internal Medicine II, University Hospital Würzburg, Würzburg, Germany; 3 National Center for Tumor Diseases (NCT), Würzburg, Germany; 4 Bavarian Cancer Research Center (BZKF), Würzburg, Germany; 5 Fraunhofer Institute for Cell Therapy and Immunology IZI, Cellular Immunotherapy, Würzburg, Germany; 6 Mildred Scheel Early Career Center, Würzburg, Germany

**Keywords:** antibody-dependent cell cytotoxicity, antibody–drug conjugates (ADCs), BCMA-targeting ADC belantamab-mafodotin, chimeric antigen receptor (CAR) T cell therapy, controllable CAR-T systems, multiple myeloma

## Abstract

Recent advances in chimeric antigen receptor (CAR) T cell therapy have transformed the treatment landscape of multiple myeloma, yet almost all patients ultimately relapse. Chromosomal 1q gains are associated with a higher risk of disease progression and poor prognosis, suggesting that CAR-T targeting of chromosome 1–encoded antigens, such as SLAMF7, may be particularly relevant in advanced disease. However, novel CAR targets raise the risk of on-target, off-tumor toxicities, underscoring the need for controllable CAR-T systems. We systematically assessed pharmacologic and antibody-based strategies to modulate CD19- and SLAMF7-directed CAR-T cells. Tyrosine-kinase inhibitor dasatinib rapidly and reversibly inhibited CAR-T activation, serving as an efficient “on/off” switch with the limitation of also inhibiting unmodified T cells. To surpass this issue, we used antibody-dependent cell cytotoxicity to inhibit CAR-T cells. However, conditioning with fludarabine/cyclophosphamide profoundly depletes NK cells, limiting antibody-dependent CAR-T clearance in patients. Moreover, as NK cells express SLAMF7, they are susceptible to fratricidal cytotoxicity by SLAMF7 CAR-T cells, further reducing this potential off-switch mechanism. To bypass this immune effector cell dependence, we developed a novel strategy using antibody–drug conjugates (ADCs). In this work, we demonstrate that the BCMA-targeting ADC belantamab-mafodotin selectively eliminates BCMA co-expressing CAR-T cells without affecting unmodified T cells. These findings suggest ADCs as a potent, effector cell-independent safety mechanism for CAR-T therapies, potentially enhancing controllability and safety in future clinical applications.

## Introduction

Recent approvals of BCMA-directed CAR-T cells have reshaped multiple myeloma (MM) treatment, yielding impressive clinical responses (1–3). However, the vast majority of MM patients eventually relapse (4), partially due to downregulation, loss or conformational changes of the B-cell maturation antigen (BCMA) (5). Genomic aberrations of the BCMA-encoding gene *TNFRSF17* (chr16p13.13) often coincide with additional chromosomal variations, including copy number gains of *SLAMF7* on chromosome 1 (chr1q) (6). Independently from BCMA-directed therapies, these chr1q gains are associated with disease progression and poor prognosis (7). Directing CAR-T cells against antigens encoded on chromosome 1 is thus a strategy to target presumably more stable antigens in potentially more advanced disease stages.

Application of CAR-T cells targeting novel tumor antigens can cause unexpected on-target, off-tumor toxicities due to antigen expression on healthy tissues (8, 9). To manage such risks, strategies to control CAR-T cell activity and persistence in case of exuberant toxicity are critical. Proposed approaches range from pharmacological drugs to genomically incorporated “suicide” genes (10). However, major disadvantages like immunogenicity, insufficient activation and toxicities, have hampered many of these approaches (11, 12). Exploring different CAR-T cell unspecific, but also specific, control strategies, we here identify antibody-drug conjugates (ADCs), monoclonal antibodies linked to cytostatic payloads that penetrate antigen-expressing cells and induce fatal damage (13), as a CAR-T cell selective depletion method independent of a functional effector cell compartment.

## Methods

### Manufacturing of CAR-T cells

**Figure 1:**
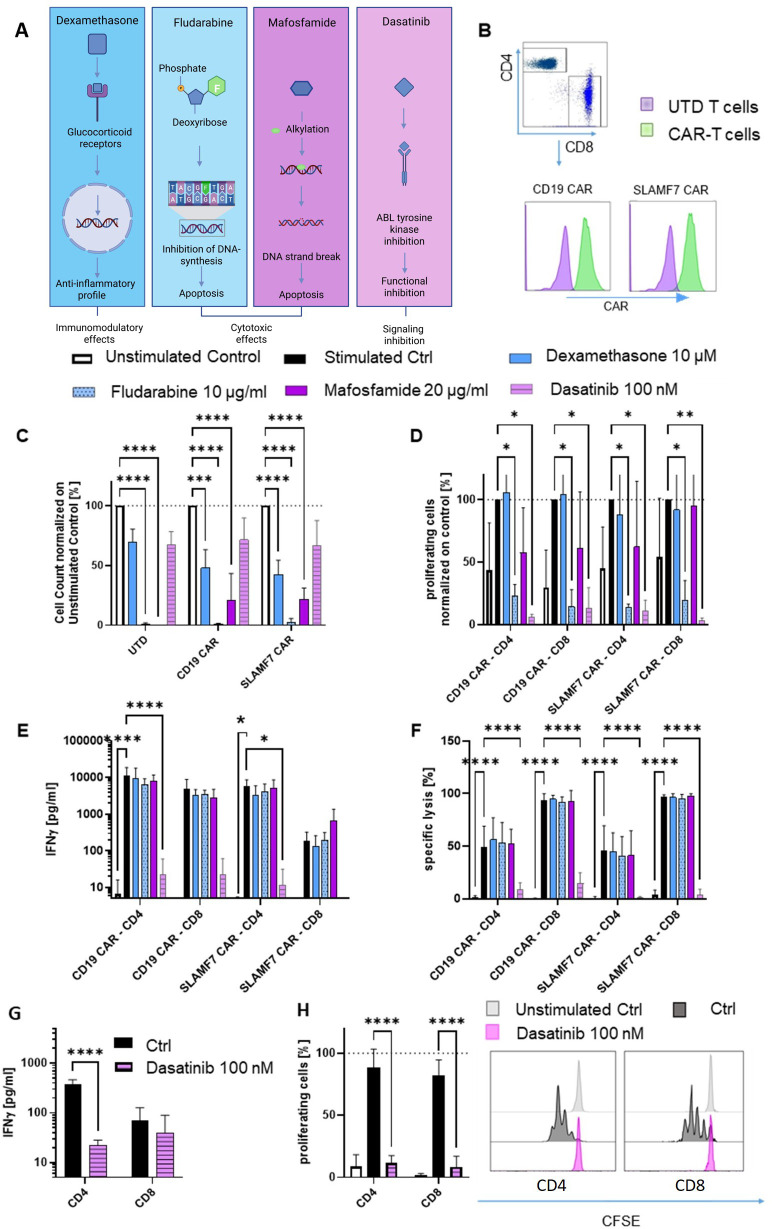
Dasatinib, but not dexamethasone, fludarabine or mafosfamide decreases CAR-T cell functionality and proliferation in an activation specific manner; **(A)** Schematic depiction of the tested drugs and their mechanisms of action; **(B)** Gating strategy of CD4 and CD8 CAR-T cells with an incorporated tEGFR marker representing CAR expression; **(C)** Unstimulated UTD cells, CD19 and SLAMF7 CAR-T cells were incubated with dexamethasone, fludarabine, mafosfamide or dasatinib at different concentrations and cell count was measured after 72 h using flow cytometry (n=3; normalized on untreated cells; CD4:CD8 1:1); **(D–F)** CAR-T cells were stimulated with K562 cells, either expressing the respective target antigen (CD19 or SLAMF7; E:T 5:1) or cocultured with K562 cells expressing an unrelated target (Unstimulated control; E:T 5:1); Proliferation was measured after 72 h (**(D)** CFSE; n=3, normalized on medium control); IFNy release (**(E)** n=3) and specific lysis of the target cells (**(F)** n=4) were measured after 24 h; **(G, H)** IFNγ release (**(G)** after 24 h; n=3) and proliferation (**(H)** CFSE after 72 h; n=3) upon stimulation with CD3/CD28 Dynabeads Human activator beads with and without dastinib; Two-way ANOVA statistic test was performed using GraphPad Prism 9; Abbreviations: CAR, Chimeric antigen receptor; Ctrl, Control; EGFRt, Truncated epidermal growth factor receptor; E:T, effector:target ratio; UTD, Untransduced. **** = P ≤ 0,0001; ***= P ≤ 0,001; ** = P ≤ 0,01; * = P ≤ 0,05.

Healthy donor peripheral blood mononuclear cells (PBMCs) were obtained from leucocyte reduction chambers provided by the Department for Transfusion Medicine of the University Hospital Würzburg. Written informed consent to participate in research protocols was given by all donors. CAR-T cells directed against SLAMF7 or CD19 were generated (**Figure 1B**) as described previously (14).

### Cell line and culture media

K562 cell line (human CML; ATCC CCL-243, USA) was cultured in RPMI-1640 medium (Thermo Fisher Scientific; 72400-054) containing 10 % fetal calf serum and 100 U/ml penicillin/streptomycin (Thermo Fisher Scientific; 15070063). Firefly luciferase (ffLuc)/GFP positive sublines additionally expressing either SLAMF7 or CD19 were generated by lentiviral transduction as previously described (15).

### Co-culture assays

SLAMF7 and CD19 CAR-T cells were exposed to therapeutically achievable concentrations of dexamethasone (10 µM), fludarabine (10 µg/ml), mafosfamide (20 µg/ml) as the active cyclophosphamide analogue, and dasatinib (100 nM; **Figure 1A**). The cells were stimulated through co-cultivation with SLAMF7- or CD19-expressing K562 cells, or were left resting for 24 h and 72 h. To investigate antibody-dependent cell cytotoxicity (ADCC) as potential CAR-T cell depletion strategy, CD19 and SLAMF7 CAR-T cells, co-expressing truncated EGFR (tEGFR), were exposed to autologous PBMCs (E:T 50:1) or natural killer (NK) cells (E:T 10:1) in the presence or absence of 50 µg/ml anti-EGFR antibody cetuximab (Erbitux^®^/Merck KGaA, Darmstadt, Germany) for 24 h. To analyze the potential of ADCs to deplete CAR-T cells, SLAMF7 CAR-T cells and unmanipulated T cells with and without co-expression of BCMA, were incubated with belantamab-mafodotin (belamaf; Blenrep^®^/GSK, London, UK) or an IgG1 isotype control at a target concentration of 50 µg/ml for 48 h and 72 h.

### Flow cytometry

Data was collected on a MACS Quant 10 (Miltenyi Biotech, Germany) and analyzed using FlowJo V10.8.1 (FlowJo LLC, USA). Cells were stained as previously described (16). CAR-T cell viability (7-AAD; Miltenyi Biotech; 130-111-568) and proliferation (CellTrace™ CFSE Cell Proliferation Kit; Thermo Fisher Scientific; C34570) were measured after 24 h and 72 h, respectively. Antibodies used in this research report were specific for BCMA-Antibodies used in this research report were specific for BCMA (Miltenyi Biotec; 130-119-152), EGFR (Cetuximab, in-house labelled), CD8 (Miltenyi Biotec; 130-110-683) and CD4 (Miltenyi Biotec; 130-114-534).

### Cytokine release analysis

After 24 h of culture, interferon y (IFNy*)* release was quantified by ELISA (ELISA MAX Deluxe Set Human IFNy; Biolegend; 430116) using the Tecan Spark (Männedorf, Switzerland).

### Statistical analysis

Statistical analysis was performed *via* Graphpad Prism V9.3.0. (GraphPad Software Inc., USA). Individual tests are indicated in the respective figure legend. P-values are represented by: **** = P ≤ 0,0001; *** = P ≤ 0,001; ** = P ≤ 0,01; * = P ≤ 0,05; ns = P > 0,05.

## Results

### Dasatinib decreases CAR-T cell functionality and proliferation in an activation specific manner

We first investigated the relevance of conventional pharmacological drugs on CAR-T cell counts by co-incubating stimulated or resting SLAMF7 and CD19 CAR-T cells with different lymphomodulating drugs, schematically categorized in **Figure 1A**. High-dose dexamethasone is known to mitigate CAR-T cell toxicities (17). In our study, high-dose dexamethasone (18) significantly reduced CAR-T cell numbers in unstimulated culture (**Figure 1C**), without impairing antigen specific CAR-T cell proliferation, IFNγ secretion, or target cell lysis (**Figures 1D-F**). Fludarabine, commonly incorporated into conditioning protocols prior to CAR-T cell administration for profound lymphodepletion (19–21), resulted in significantly reduced T cell numbers regardless of cell-based antigen stimulation across conditions, while antigen-specific IFNγ secretion and target cell lysis remained unaffected (**Figures 1C-F**). Mafosfamide, the biochemically active counterpart of cyclophosphamide, the second component of standard lymphodepletion regimens, exhibited similar effects to fludarabine, though antigen-specific stimulation was able to restore CAR-T cell proliferation (**Figure 1D**). For all three drugs, the effects were independent of CAR specificities. The tyrosine kinase inhibitor dasatinib, that blocks CAR downstream signaling by inhibiting CD3ζ phosphorylation, has been proposed as functional CAR-T cell “off-switch” (22). Here, we observed no effects of dasatinib on resting T cells, however complete inhibition of proliferation, IFNγ secretion and target cell lysis upon cell-based target antigen stimulation (**Figures 1C-F**). Importantly, in T cells stimulated in an antigen independent manner, secretion and proliferation capacities were similarly affected by dasatinib (**Figures 1G, H**).

### Depletion of CAR-T cells through antibody-dependent cell cytotoxicity depends on functional natural killer cells

**Figure 2:**
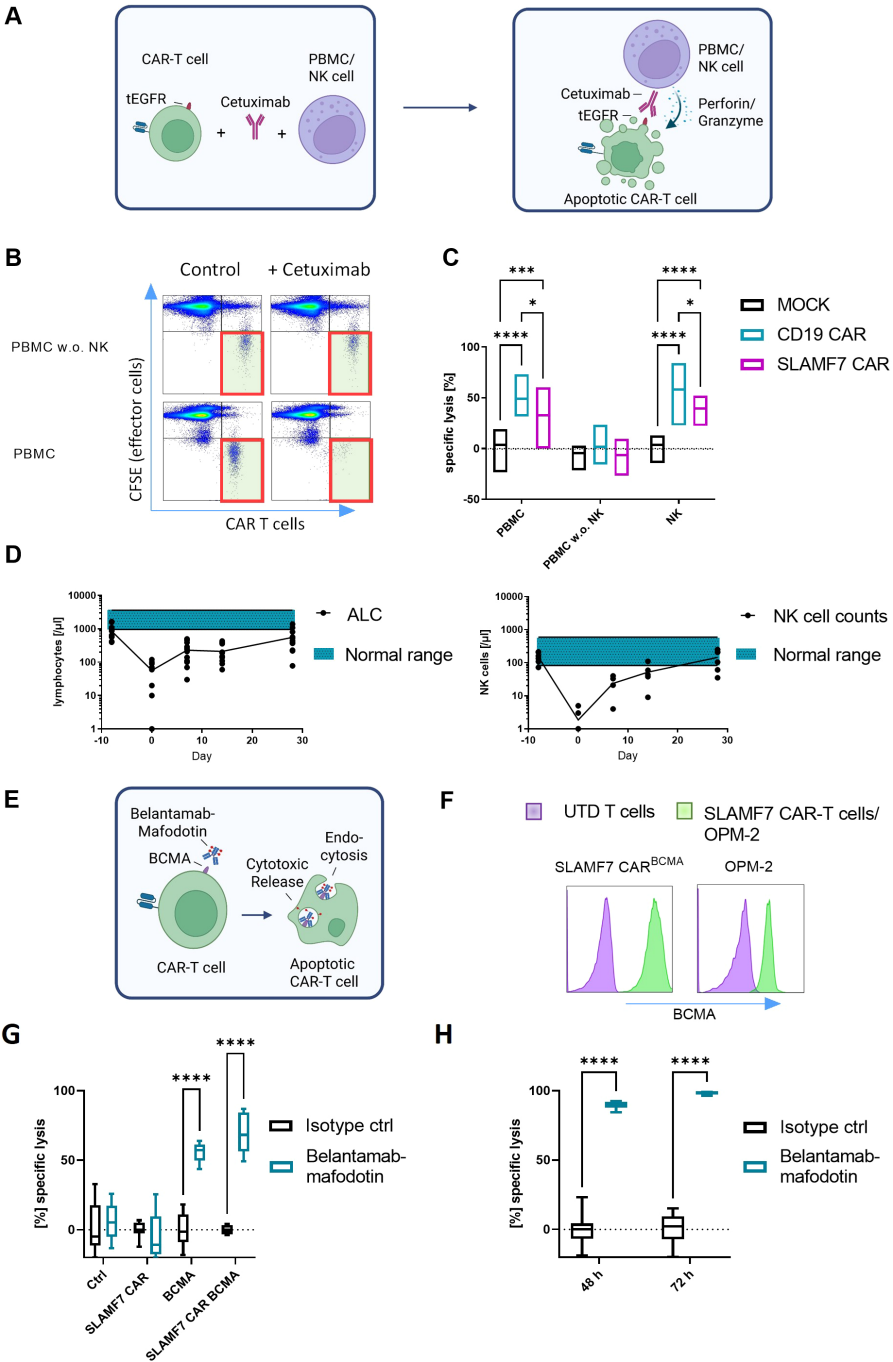
Efficiency of ADCC and ADC on SLAMF7 and CD19 CAR-T cells *in vitro*; **(A)** Schematic depiction of EGFR-specific ADCC; **(B, C)** CD19 and SLAMF7 CAR-T cells (CD4:CD8 1:1) were incubated with EGFR antibody cetuximab [50 µg/ml] and cocultured with effector cells (CFSE stained, PBMC w./w.o. NK cells, E:T 50:1 and NK cells, E:T 10:1) for 24 h, CAR-T cell elimination through ADCC is dependent on NK cell availability and CAR-T cell construct, Representative Flow cytometry plots **(B)** and statistical analysis ((C) n=3) are shown; **(D)** Lymphocyte (n=12) and NK cell (n=8) counts are reduced after lymphodepleting chemotherapy (d-5, -4 and -3) and only gradually recover; **(E)** Schematic depiction of the ADC assay; **(F)** BCMA expression of targeted cells via flow cytometry, **(G, H)** Cells expressing BCMA or not, were incubated with BCMA targeting ADC belantamab-mafodotin [50 µg/ml]; SLAMF7 CAR-T cells, UTD ctrl T cells (72 h; **(G)**) and the human MM cell line OPM-2 (48 h & 72 h; **(H)**) are shown; Control antibody: Human IgG1 Isotype Control; n=3 independent donors; Each experiment was performed in triplicates; Two-way ANOVA statistic test was performed using GraphPad Prism 9; Abbreviations: ADC, Antibody-drug conjugate; ADCC, Antibody-dependent cell cytotoxicity; ALC, Absolute Lymphocyte Count; CAR, Chimeric antigen receptor; Ctrl, Control; MM, Multiple myeloma; SLAMF7, SLAM Family member 7 (CD319); UTD, Untransduced. **** = P ≤ 0,0001; *** = P ≤ 0,001; * = P ≤ 0,05.

As a more CAR-T cell selective approach, we evaluated antibody-mediated depletion strategies (**Figure 2A**). To apply ADCC-based CAR-T cell elimination in our setting, we exposed CD19 and SLAMF7 CAR-T cells, co-expressing tEGFR, to autologous PBMCs or NK cells in the presence or absence of cetuximab. While we found significant CAR-T cell depletion, unmanipulated T cells were entirely spared (**Figures 2B, C**). However, depletion did not occur when NK cells were removed from the PBMCs. The presence of the non-NK cell PBMC populations, including monocytes, had no effect on the viability of the CAR-T cells, underlining NK cell dependency of ADCC. Interestingly, SLAMF7 CAR-T cells were significantly less sensitive to cetuximab-mediated ADCC than CD19 CAR-T cells. To characterize the lymphocyte compartment following conditioning therapy with fludarabine/cyclophosphamide prior to CAR-T cell administration, we retrospectively analyzed data from patients included into phase I CAR-T cell trials at our institution between 2020 and 2024 (NCT04499339, NCT04230265). Patients consistently experienced grade 3/4 lymphopenia at the time of CAR-T cell transfer until day +28. Notably, NK cell counts dropped dramatically, particularly within the first week post CAR-T cell infusion, when NK cell counts were below 10 % of the median in healthy donors (**Figure 2D**), highlighting the need for effector cell-independent depletion strategies.

### Antibody-drug conjugate belantamab-mafodotin eliminates CAR-T cells in an effector cell-independent manner

ADCs are a tool for effector cell-independent targeted therapy. As the clinical development of EGFR-directed ADCs is yet in its infancy (11), we replaced the tEGFR marker in our CAR-T cells with BCMA, thereby enabling the targeting of the CAR-T cells with the approved BCMA-directed ADC belamaf (12). Indeed, co-incubation with belamaf resulted in significant depletion of BCMA^+^ CAR-T cells despite the absence of immune effector cells, while tEGFR^+^ CAR-T cells and unmanipulated T cells were fully spared (**Figures 2E-G**). Belamaf equally depleted BCMA^+^ MM cells *in vitro* (**Figure 2H**).

## Discussion

This research report explores various strategies to regulate CAR-T cell activity. Known pharmacologic drugs with various effects, clinically approved for different hematological cancers, were hypothesized to regulate either on a functional level or modify CAR-T cell viability. Our *in vitro* results suggest only limited direct effects of dexamethasone on activated T cells. In comparison, fludarabine and mafosfamide demonstrate potent depletion of CAR-T cells, while effector functions in remaining CAR-T cells are largely preserved. The depletion capacity of mafosfamide is notably diminished by stimulation of CAR-T cells, rendering it even less suitable in case of uncontrolled CAR-T cell activation. Dasatinib, on the other hand, induces rapid and complete inhibition of proliferation, IFNγ secretion, and target cell lysis in response to antigen stimulation, indicating its potential as a swift mechanism to suppress CAR-T cell activity without affecting resting T cells. However, since dasatinib affects all stimulated T cells indiscriminately, it does not offer CAR specificity, which might pose the patients at increased risk for infections. Hence, the development of CAR-selective approaches to spare unmanipulated host T cells is essential.

One such CAR-selective-approach relies on the use of monoclonal antibodies like cetuximab, to target surface markers co-expressed on CAR-T cells, like tEGFR (23). This approach was found to potently deplete CD19 CAR-T cells in murine models, however clinical application has not yet been demonstrated. Any interference with EGFR signaling in the CAR-T cells is not expected, owing to the intracellular truncation of the EGFR serving as CAR transduction marker in this system. Our findings show the feasibility of ADCC-mediated CAR-T depletion *in vitro.* However, we hypothesized that lymphodepletion, especially NK cell reduction induced by conditioning regiments, can compromise the CAR-T cell elimination capacity of monoclonal antibodies by reducing ADCC. Our results support this hypothesis by highlighting the reliance of ADCC on NK cells. While the ADCC strategy precisely targets CAR-T cells and spares unrelated cells, a high dependence on an intact NK cell compartment for the execution is shown. This dependence likely also explains why SLAMF7 CAR-T cells were significantly less sensitive to cetuximab-mediated ADCC than CD19 CAR-T cells, as fratricidal elimination of SLAMF7^+^ NK cells may occur before cetuximab-induced CAR-T cell depletion can take place.

The ADC belamaf elegantly overcomes the aforementioned limitations. By demonstrating CAR selectivity, it spares unmanipulated as well as resting T cells, thereby reducing the risk of infection compared to non-selective approaches. Moreover, ADCs act effectively in a cell-independent manner. Clearly, the concept is not limited to BCMA as target, but rather applicable for any antigen targetable by FDA/EMA approved ADCs. Not only does this circumvent issues like antigen-loss and clonal selection after BCMA CAR-T cell treatment in MM, but it also opens up the applicability of this approach to various cancer entities and CAR-T cell designs. Together, these observations highlight the potential of ADCs to specifically and reliably deplete CAR-T cells, even in the absence of functional immune effector cells.

Yet, our study contains several limitations. First, this strategy requires a suitable ADC product and target. While various ADCs have been FDA/EMA-approved in the last decade, ADCs can also cause toxicities and especially a premature release of the cytostatic payload may result in systemic adverse events (24). However, for CAR-T cell depletion, we expect a single administration to suffice, limiting the probability of toxicities associated with repetitive dosing. Second, most ADCs are tubulin inhibitory drugs, that mainly act on proliferating cells by arresting them at the G2/M phase (25). The combinatorial use with other drugs that inhibit proliferation might hamper their potency further. However, in cases of uncontrollable CAR-T cell toxicities, rapid expansion of excessively activated CAR-T cells is characteristic, making tubulin-inhibiting ADCs a viable strategy to control CAR-T cell toxicity. Third, this proof-of-concept idea has yet to be validated in preclinical *in vivo* systems, before clinical translation becomes tangible.

In aggregate, our findings support the use of ADCs as an efficient and specific method to target CAR-T cells in an immune effector cell-independent manner. The incorporation of defined surface markers makes this approach adaptable for a range of applications. As tumor-exclusive CAR targets are rare, the incorporation of specific ADCs for CAR-T cell depletion in case of uncontrollable toxicity has the potential to reduce barriers and improve safety for the clinical translation of CAR-T cell products directed against novel target antigens.

## Data Availability

The original contributions presented in the study are included in the article/supplementary material. Further inquiries can be directed to the corresponding author.

## References

[1] Hansen DK Lu X Puglianini OC Sorensen S Usmani SZ Zhang E . Cost-per-responder analysis of patients with lenalidomide-refractory multiple myeloma receiving ciltacabtagene autoleucel in CARTITUDE-4. Front Immunol. (2024) 15:1408892. doi: 10.3389/fimmu.2024.1408892, PMID: 39234256 PMC11372240

[2] Rodriguez-Otero P Ailawadhi S Arnulf B Patel K Cavo M Nooka AK . Ide-cel or standard regimens in relapsed and refractory multiple myeloma. N Engl J Med. (2023) 388:1002–14. doi: 10.1056/NEJMoa2213614, PMID: 36762851

[3] San-Miguel J Dhakal B Yong K Spencer A Anguille S Mateos MV . Cilta-cel or standard care in lenalidomide-refractory multiple myeloma. N Engl J Med. (2023) 389:335–47. doi: 10.1056/NEJMoa2303379, PMID: 37272512

[4] Xu J Wang BY Yu SH Chen SJ Yang SS Liu R . Long-term remission and survival in patients with relapsed or refractory multiple myeloma after treatment with LCAR-B38M CAR T cells: 5-year follow-up of the LEGEND-2 trial. J Hematol Oncol. (2024) 17:23. doi: 10.1186/s13045-024-01530-z, PMID: 38659046 PMC11040812

[5] Da Via MC Dietrich O Truger M Arampatzi P Duell J Heidemeier A . Homozygous BCMA gene deletion in response to anti-BCMA CAR T cells in a patient with multiple myeloma. Nat Med. (2021) 27:616–9. doi: 10.1038/s41591-021-01245-5, PMID: 33619368

[6] Lee H Ahn S Maity R Leblay N Ziccheddu B Truger M . Mechanisms of antigen escape from BCMA- or GPRC5D-targeted immunotherapies in multiple myeloma. Nat Med. (2023) 29:2295–306. doi: 10.1038/s41591-023-02491-5, PMID: 37653344 PMC10504087

[7] Schmidt TM Fonseca R Usmani SZ . Chromosome 1q21 abnormalities in multiple myeloma. Blood Cancer J. (2021) 11:83. doi: 10.1038/s41408-021-00474-8, PMID: 33927196 PMC8085148

[8] Morgan RA Yang JC Kitano M Dudley ME Laurencot CM Rosenberg SA . Case report of a serious adverse event following the administration of T cells transduced with a chimeric antigen receptor recognizing ERBB2. Mol Ther. (2010) 18:843–51. doi: 10.1038/mt.2010.24, PMID: 20179677 PMC2862534

[9] Thistlethwaite FC Gilham DE Guest RD Rothwell DG Pillai M Burt DJ . The clinical efficacy of first-generation carcinoembryonic antigen (CEACAM5)-specific CAR T cells is limited by poor persistence and transient pre-conditioning-dependent respiratory toxicity. Cancer Immunol Immunother. (2017) 66:1425–36. doi: 10.1007/s00262-017-2034-7, PMID: 28660319 PMC5645435

[10] Lu L Xie M Yang B Zhao WB Cao J . Enhancing the safety of CAR-T cell therapy: Synthetic genetic switch for spatiotemporal control. Sci Adv. (2024) 10:eadj6251. doi: 10.1126/sciadv.adj6251, PMID: 38394207 PMC10889354

[11] Carneiro BA Papadopoulos KP Strickler JH Lassman AB Waqar SN Chae YK . Phase I study of anti-epidermal growth factor receptor antibody-drug conjugate serclutamab talirine: Safety, pharmacokinetics, and antitumor activity in advanced glioblastoma. Neurooncol Adv. (2023) 5:vdac183. doi: 10.1093/noajnl/vdac183, PMID: 36814898 PMC9940695

[12] Markham A . Belantamab mafodotin: first approval. Drugs. (2020) 80:1607–13. doi: 10.1007/s40265-020-01404-x, PMID: 32936437

[13] Sievers EL Senter PD . Antibody-drug conjugates in cancer therapy. Annu Rev Med. (2013) 64:15–29. doi: 10.1146/annurev-med-050311-201823, PMID: 23043493

[14] Wang Q He F He W Huang Y Zeng J Zi F . A transgene-encoded truncated human epidermal growth factor receptor for depletion of anti- B-cell maturation antigen CAR-T cells. Cell Immunol. (2021) 363:104342. doi: 10.1016/j.cellimm.2021.104342, PMID: 33765541

[15] Prommersberger S Hudecek M Nerreter T . Antibody-based CAR T cells produced by lentiviral transduction. Curr Protoc Immunol. (2020) 128:e93. doi: 10.1002/cpim.93, PMID: 32150338

[16] Feucht J Sun J Eyquem J Ho YJ Zhao Z Leibold J . Calibration of CAR activation potential directs alternative T cell fates and therapeutic potency. Nat Med. (2019) 25:82–8. doi: 10.1038/s41591-018-0290-5, PMID: 30559421 PMC6532069

[17] Neelapu SS Tummala S Kebriaei P Wierda W Gutierrez C Locke FL . Chimeric antigen receptor T-cell therapy - assessment and management of toxicities. Nat Rev Clin Oncol. (2018) 15:47–62. doi: 10.1038/nrclinonc.2017.148, PMID: 28925994 PMC6733403

[18] Brown S Pawlyn C Tillotson AL Sherratt D Flanagan L Low E . Bortezomib, vorinostat, and dexamethasone combination therapy in relapsed myeloma: results of the phase 2 MUK four trial. Clin Lymphoma Myeloma Leuk. (2021) 21:154–61 e3. doi: 10.1016/j.clml.2020.11.019, PMID: 33478922

[19] Teitz-Tennenbaum S Li Q Davis MA Wilder-Romans K Hoff J Li M . Radiotherapy combined with intratumoral dendritic cell vaccination enhances the therapeutic efficacy of adoptive T-cell transfer. J Immunother. (2009) 32:602–12. doi: 10.1097/CJI.0b013e3181a95165, PMID: 19483649 PMC2743975

[20] Hirayama AV Gauthier J Hay KA Voutsinas JM Wu Q Gooley T . The response to lymphodepletion impacts PFS in patients with aggressive non-Hodgkin lymphoma treated with CD19 CAR T cells. Blood. (2019) 133:1876–87. doi: 10.1182/blood-2018-11-887067, PMID: 30782611 PMC6484391

[21] Ong SY Pak S Mei M Wang Y Popplewell L Baird JH . Bendamustine lymphodepletion is a well-tolerated alternative to fludarabine and cyclophosphamide lymphodepletion for axicabtagene ciloleucel therapy for aggressive B-cell lymphoma. Am J Hematol. (2023) 98:1751–61. doi: 10.1002/ajh.27069, PMID: 37668287 PMC10666914

[22] Mestermann K Giavridis T Weber J Rydzek J Frenz S Nerreter T . The tyrosine kinase inhibitor dasatinib acts as a pharmacologic on/off switch for CAR T cells. Sci Transl Med. (2019) 11(499):eaau5907. doi: 10.1126/scitranslmed.aau5907, PMID: 31270272 PMC7523030

[23] Paszkiewicz PJ Frassle SP Srivastava S Sommermeyer D Hudecek M Drexler I . Targeted antibody-mediated depletion of murine CD19 CAR T cells permanently reverses B cell aplasia. J Clin Invest. (2016) 126:4262–72. doi: 10.1172/JCI84813, PMID: 27760047 PMC5096899

[24] Taylor RP Lindorfer MA . Antibody-drug conjugate adverse effects can be understood and addressed based on immune complex clearance mechanisms. Blood. (2024) 144:137–44. doi: 10.1182/blood.2024024442, PMID: 38643493

[25] Riccardi F Dal Bo M Macor P Toffoli G . A comprehensive overview on antibody-drug conjugates: from the conceptualization to cancer therapy. Front Pharmacol. (2023) 14:1274088. doi: 10.3389/fphar.2023.1274088, PMID: 37790810 PMC10544916

